# Optimization of Deep Eutectic Solvent Extraction of Phenolic Acids and Tannins from *Alchemilla vulgaris* L.

**DOI:** 10.3390/plants11040474

**Published:** 2022-02-09

**Authors:** Martina Jakovljević Kovač, Stela Jokić, Igor Jerković, Maja Molnar

**Affiliations:** 1 Faculty of Food Technology Osijek, Josip Juraj Strossmayer University of Osijek, Franje Kuhača 18, 31000 Osijek, Croatia; mjakovljevic@ptfos.hr (M.J.K.); sjokic@ptfos.hr (S.J.); 2 Faculty of Chemistry and Technology, University of Split, Ruđera Boškovića 35, 21000 Split, Croatia

**Keywords:** *Alchemilla vulgaris* L., optimization, gallic acid, ellagic acid, hydrolyzable tannins, extraction

## Abstract

*Alchemilla vulgaris* L. is a good source of antioxidant components with an emphasis on phenolic acids and tannins. In this study, gallic acid, ellagic acid, and hydrolyzable tannins (HT) were extracted from this plant with different deep eutectic solvents (DESs), varying the amount of added H_2_O, temperature and extraction time. Seventeen DESs (*n* = 3) were used for the extraction, of which choline chloride:urea (1:2) proved to be the most suitable. The selection of the best solvent was followed by the examination of the influence of the extraction type and parameters using response surface methodology (RSM). Gallic acid content was in the range of 0.00–1.89 µg mg^−1^, ellagic acid content was 0.00–12.76 µg mg^−1^ and hydrolyzable tannin (HT) content was 3.06–181.26 µgTAE mg^−1^, depending on the used technique and the extraction conditions. According to the results, extraction by stirring and heating was the most suitable since the highest amounts of gallic acid, ellagic acid, and HT were extracted, and the obtained optimal values using response surface methodology (RSM) are confirmed by experimentally obtained values.

## 1. Introduction

Nature has always been an inexhaustible source of biologically active compounds that have long been used in the form of folk medicine to treat various diseases and health conditions [1]. Medicinal plants are an important source of biologically active components, leading to the numerous possibilities of their application in the form of medical treatments or as novel drug formulations [2]. Numerous studies support the hypothesis that plant secondary metabolites, with an emphasis on phenols, which are bioactive compounds, may play an important role in the oxidation processes. Their role is manifested through the reduction of the detrimental effect of the imbalance between the formation of enzymatic and non-enzymatic antioxidants and the excessive amount of free radicals formed in the process of oxidative stress [3]. As a result of antioxidant activity, phenolic components show a number of beneficial health effects, such as anti-inflammatory and anticancerogenic activities [4,5].

One of the well-known medicinal plants is the common lady’s mantle (*Alchemilla vulgaris* L.)*,* a perennial herbaceous plant that belongs to the Rosaceae family. It is commonly found throughout almost all of Europe, including larger areas of the European territory of Russia and Siberia [6] as well as in western Asia and North America [7]. *Alchemilla vulgaris* L. has been used in folk medicine for many years to treat a number of conditions, such as skin (ulcers, wounds, eczema), digestive (diarrhea), and gynecological disorders (heavy menstrual flow, menorrhagia, and dysmenorrhea) [8,9,10]. The effectiveness of the use of *Alchemilla vulgaris* L. for these conditions is reflected in a number of biological activities, such as antiviral, antioxidant, antiproliferative, and antibacterial activity [11,12,13].

According to the research, the aerial parts of *A. vulgaris* contain numerous phenolic components with an emphasis on phenolic acids (ellagic acid, gallic, and caffeic acids), flavonoids (quercetin, isoquercetin, rutin, avicularin, and tiliroside), and tannins agrimoniin, pedunculagin, and laevigatin F [14,15]. Phenolic components from this plant are usually extracted using conventional organic solvents. Duckstein et al. (2012) used acetone/water to extract different ellagitannins and gallic and chlorogenic acids from *A. vulgaris* leaves (including stalks) [7], while Boroja et al. (2018) extracted phenolic compounds from ground parts and roots by maceration in methanol [2]. Bioactive compounds from dry plant material could be extracted using other solvents, like 80% methanol, 70% ethanol, 70% ethylacetate and distilled water by 24 h maceration, where ethylacetate was shown to be the most effective in the extraction of phenolic compounds (gallic acid, caffeic acid, ferulic acid, quercetin, catechin, kaempferol), also possessing the highest antioxidant activity [1].

The importance of phenolic components is increasingly recognized nowadays, and consequently, there has been increased development of their new extraction and isolation methods [16]. The most important difference between conventional and modern extraction techniques is better efficiency in shorter extraction time of the latter ones.

In recent years, deep eutectic solvents (DESs), first mentioned by Abbott et al. [17,18] are used in different areas, including the extraction of phenolic components. The reasons for the increasing use of DESs in the field of extraction are primarily related to the low cost of the starting components and the ease of preparation of the solvents, which are biodegradable with no or low toxicity. On the other hand, numerous studies have shown that they have better extraction efficiency compared to the conventional solvents, as they dissolve lignocelluloses better, destroying the cell structure and achieving better mass transfer [19,20,21,22]. It is important to note that DESs can be prepared from many starting components in different proportions, making them design solvents with tunable properties, which as a result, also show different functionality and solubility for the components. Therefore, the application of DESs consisting of appropriate molar ratios and combinations of components can lead to improved solubility and extraction efficiency for the desired components. However, despite the many advantages, DESs viscosity is their main disadvantage, while the low vapor pressure can be considered as both advantage and disadvantage. Both above-mentioned characteristics need to be taken into consideration as they complicate the extraction process as well as the isolation of the desired components [23].

Choline chloride (ChCl) is very often used as a hydrogen bond acceptor (HBA) for the preparation of DESs, as it is cheap, available, biodegradable, as well as a non-toxic quaternary ammonium salt that can form DESs with other non-toxic components such as amines, sugars, sugar alcohols, and carboxylic acid used as hydrogen bond donor (HBD) [17,18].

In recent years, DESs have been increasingly used for the extraction of phenolic components, with an emphasis on phenolic acids, flavonoids, lignans, coumarins, stilbenes, tannins, and anthocyanins. Choline chloride-based DESs have shown to be more efficient in the extraction of phenolic components including gallic acid compared to betaine-based DESs and proline-based DESs. Looking at choline chloride-based DESs, the solvents with polyol have been shown to be very effective in the extraction of phenolic acids [24].

To the best of our knowledge, there are no published papers on the extraction and optimization of gallic acid, ellagic acid and hydrolyzable tannins (HT) content using DESs from *Alchemilla vulgaris* L. Accordingly, the objectives of this study were focused on (1) finding the most suitable choline chloride-based DESs for the extraction of gallic and ellagic acid as well as HT. Subsequently, (2) the influence of the selected extraction parameters on the content of gallic acid and ellagic acid as well as HT in the extracts was examined using high-performance liquid chromatography (HPLC) and spectrophotometry. In addition (3), the most suitable extraction technique was selected. The optimal extraction conditions (4) determined by response surface methodology (RSM) for desired components were experimentally tested to confirm the effectiveness of the model.

## 2. Results and Discussion

Since DESs differ not only in physicochemical properties, depending on the components, but also in the ability to dissolve and extract certain components, several different solvents need to be tested to determine the most suitable solvent for extracting the desired components. In this case, 17 different DESs (Table 1) were used for the extraction of gallic and ellagic acids as well as HT.

### 2.1. Influence of DES Type on the Obtained Amount of Gallic and Ellagic Acids and HT

**Figure 1:**
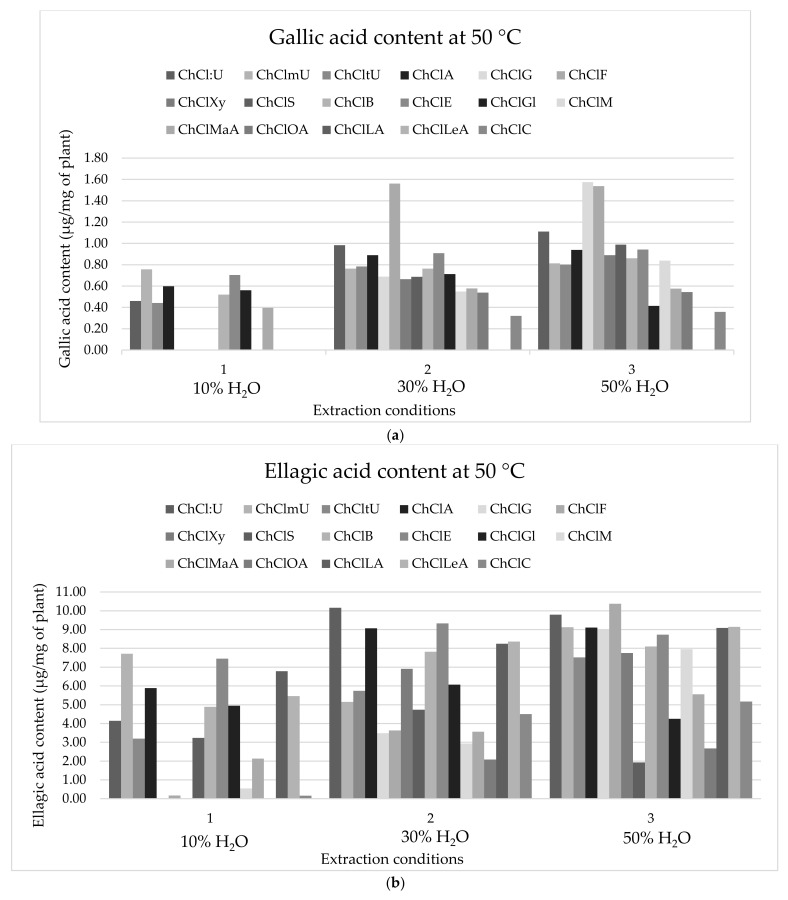

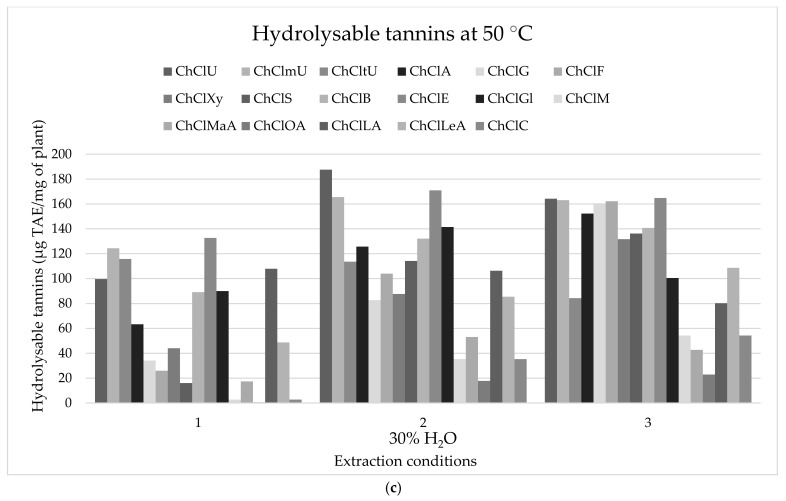
(**a**) Gallic acid content, (**b**) ellagic acid content, and (**c**) HT content in the extracts obtained with different DESs using stirring and heating depending on the temperature and H_2_O content (*n* = 3). The columns represent a certain amount of gallic acid, ellagic acid, and hydrolyzable tannins in the extracts where the color of the column indicates the applied DES according to the legend and Table 1.

As can be seen from Figure 1, DESs differed greatly in their ability and efficiency for the extraction of gallic acid, ellagic acid, and HT. In addition to the influence of HBD, the amount of desired components was also affected by the amount of added water due to the influence on the viscosity that affects the mass transfer, thus, affecting the extraction process. Although the addition of water can decrease the viscosity, nevertheless, an excessive amount of water can decrease the interactions between the components of DESs as well as the interactions between DESs and desired components. The temperature also influenced a decrease in DES’s viscosity [25]. For these reasons, the solvent screening was performed at 50 °C with three different amounts of added water (10, 30 and 50% (*v*/*v*)).

As seen from Figure 1, only some of the 17 tested DESs were efficient in the extraction of gallic acid. At the lowest percentage of added water (10% *v*/*v*), certain DESs had a very high viscosity, which affected the low or non-existent gallic acid yield. In this case, the most effective solvents were shown to be choline chloride:methylurea (1:3) and choline chloride:ethane-1,2-diol (1:2). The increased percentage of water to 30% (*v*/*v*) led to the increase in gallic acid yield in the obtained extracts, mainly due to a decrease in solvent viscosity, which facilitated the mass transfer. In this case, the most effective solvents were choline chloride:fructose (1:1) and choline chloride:urea (1:2) with 30% addition of water (*v*/*v*). Furthermore, increasing added water to 50% (*v*/*v*) resulted not only in a slight increase in gallic acid yield, but also with the number of effective DESs. Therefore, in this case, in addition to the mentioned solvents, DES choline chloride:glucose (1:1) with 50% water (*v*/*v*) also proved to be effective. Interestingly, for choline chloride:fructose (1:1) and choline chloride:glycerol (1:2), there was a decrease in the yield of gallic acid with an increase in water content from 30% to 50%, while in choline chloride:urea (1:2) with 50% addition of water (*v*/*v*) there was an increase in the yield of gallic acid compared to the addition of 30% water (*v*/*v*). It is important to note that the addition of water not only reduced the viscosity, but also affected other physicochemical properties and the structure of DESs, so in the case of these DESs, it is possible that increasing water content up to 50% significantly affects the properties and structure of DESs thereby changing their extraction efficiency. On the other hand, the application of DESs with carboxylic acids as HBDs, shows a lower yield of gallic acid in the extracts, while with the application of choline chloride:lactic acid (1:2) and choline chloride:levulinic acid (1:2) gallic acid was not extracted. Cao et al. [26] showed that the optimal solvent for the extraction of phenolic acids from Rattan (*Calamoideae faberii*), including gallic acid, was choline chloride:ethane-1,2-diol (1:3) with the optimum volume ratio of DES:H_2_O 6:4. In addition, gallic acid was extracted from *Ginkgo biloba* leaves using DESs, with the most suitable solvents, of those prepared with choline chloride, such as choline chloride:pentane-1,5-dioic acid (1:1), choline chloride:citric acid (1:1), choline chloride:glycerol (1:2), or choline chloride:ethane-1,2-diol (1:2) [27].

A similar trend was observed in the extraction of ellagic acid using DESs, although for ellagic acid, all 17 DESs were effective (Figure 1). The lowest yield was achieved with the addition of 10% water (*v*/*v*) with the most effective solvents being the same as for the extraction of gallic acid, namely choline chloride:methylurea (1:3) and choline chloride:ethane-1,2-diol (1:2). Increasing the percentage of water to 30% (*v*/*v*) lead to an increase in ellagic acid yield in the extract, with choline chloride:urea (1:2), choline chloride:acetamide (1:2) and choline chloride:ethane-1,2-diol (1:2) with 30% water (*v*/*v*) being the most effective solvents. A further increase in water to 50% (*v*/*v*) lead not only to a slight increase in content of ellagic acid but also to a change in the efficiency of DESs in the context of ellagic acid yield. Therefore, the most efficient DESs for ellagic acid extraction were choline chloride:urea (1:2) with 50% water (*v*/*v*) and choline chloride:fructose (1:1) with 50% water (*v*/*v*). In contrast to the extraction of gallic acid, for the extraction of ellagic acid, no lower yield is observed using DESs with carboxylic acids. Moreover, at 30 and 50% added water (*v*/*v*) choline chloride:lactic acid (1:2) and choline chloride:levulinic acid (1:2) were shown to be effective. According to Rajha et al. [28], DESs such as glycerol:glycine (3:1) and glycerol:urea (1:1) proved to be the most suitable for the extraction of gallic and ellagic acid. Among seven DESs, depending on the plant material, the most efficient DESs with two components were choline chloride:glycerol (1:2), choline chloride:butane-1,4-diol (1:5). The authors also demonstrated the impact of three-component DESs, of which choline chloride:glycolic acid:oxalic acid proved to be the most effective for ellagic acid extraction (1:1.7:0.3). According to the above, it is obvious that the efficiency of DESs in the extraction also depends on the plant material used [29].

For hydrolyzable tannin (HT) extraction, it was observed that they could be extracted using all 17 DESs, with different yields depending on DESs used and the percentage of water added. The obtained results follow the trend of extraction of gallic and ellagic acid, which is not surprising given that hydrolyzable tannins include gallotannins and ellagitannins which contain gallic and ellagic acid in their composition. At 10% addition of water in DESs (*v*/*v*) it was noticed that the content of extracted HT is lower in relation to higher percentages of added water (30 and 50% (*v*/*v*)), whereby according to the results so far, the most effective solvents were choline chloride:methylurea (1:3) and choline chloride:ethane-1,2-diol (1:2). With the addition of 30% water (*v*/*v*) to the DESs, extracted HT content increase, especially with the use of choline chloride:urea (1:2) and choline chloride:ethane-1,2-diol (1:2). With 50% water added, the amount of HT extracted is lower than with 30% water. In addition, several different DESs have been shown to be effective, using choline chloride:urea (1:2), choline chloride:methylurea (1:3), choline chloride:fructose (1:1), and choline chloride:ethane-1,2-diol (1:2).

According to the obtained results, choline chloride:urea (1:2) DES was chosen for further extractions, using different extraction techniques and optimization. This solvent was chosen as it is composed of naturally occurring components that are inexpensive, readily available, and in combination form a non-toxic and biodegradable solvent [30]. In addition, choline chloride:urea (1:2) is not very viscous solvent, which allows easier handling and better mass transfer during the extraction process even at lower temperatures.

### 2.2. Influence of Different Extraction Methods on the Obtained Amount of Gallic and Ellagic Acids and Hydrolyzable Tannins

After the optimal DES was selected, the influence of different extraction techniques was examined. Stirring and heating, ultrasonic-assisted extraction (UAE) and mechanochemical extraction (MCE) using a ball mill are very often used for the extraction using DESs, so these techniques were tested here in terms of gallic acid, ellagic acid, and HT yield. All three techniques led to improved mass transfer and sped up the diffusion of compounds. When mixing and heating on a magnetic mixer, the improvement of mass transfer and diffusion occurs due to the action of a magnet that continuously mixes the contents, i.e., the plant material and the solvent. MCE works on the same principle, whereby, in this case, the glass beads move at a certain speed and thus lead to the rupture of the plant cell and to the improvement of mass transfer. In the case of UAE, the acoustic cavitation phenomenon led to the disruption of cell walls leading to better mass transfer.

According to the results in Table 2 and Table 3, it was seen that the highest yields of components were achieved by applying stirring and heating. This could be explained by reduced viscosity by heating and then better mass transfer due to continuous mixing of plant material and solvent leading to plant cell rupture and transition of components in the solvent. The application of UAE provides lower yields of components compared to stirring and heating, possibly due to the lack of mixing, which achieves better mass transfer, especially in more viscous solvents. For MCE, the utilization of the glass beads led to the mixing of plant material and solvents, but since such extraction was done in a shorter time to be able to regulate the extraction temperature, the total yields were lower compared to other techniques. However, if we compare the obtained results with MCE in Run 13 (3 min, 5 m/s and 30% water (*v*/*v*)) with Run 8 for mixing and heating and UAE (30 min, 30 and 30% water (*v*/*v*)) we observed that a larger amount of the desired components was obtained by applying MCE in only 3 min of the extraction compared to 30 min in other extraction techniques.

Therefore, the most suitable technique for the extraction of gallic acid, ellagic acid, and hydrolyzed tannins using choline chloride:urea (1:2) was mixing and heating.

### 2.3. Influence of Different DES Extraction Parameters on the Obtained Amount of Gallic and Ellagic Acids and Hydrolyzable Tannins

The influence of water addition in DES (*v*/*v*), extraction temperature or vibration speed and extraction time on the efficiency of choline chloride:urea (1:2) in the extraction of gallic acid, ellagic acid, and HT was examined by different extraction techniques. It was observed that gallic acid content was 0.00–1.89 µg mg^−1^ using stirring and heating, 0.00–1.14 µg mg^−1^ using UAE, and 0.00–1.02 µg mg^−1^ using MCE, depending on the parameters used. The content of ellagic acid in the extract obtained by mixing and heating varied in the range 0.74–12.76 µg mg^−1^, obtained by UAE in the range 0.08–10.87 µg mg^−1^ and by MCE in the range 0.00–7.07 µg mg^−1^ depending on the parameters used. The content of hydrolyzable tannins in the extracts obtained by mixing and heating varied in the range 69.59–181.26 µgTAE mg^−1^, in the extracts obtained by UAE in the range 18.53–136.39 µgTAE mg^−1^, and in the extracts by MCE in the range 3.06–115.26 µgTAE mg^−1^, depending on the parameters used.

**Figure 2:**
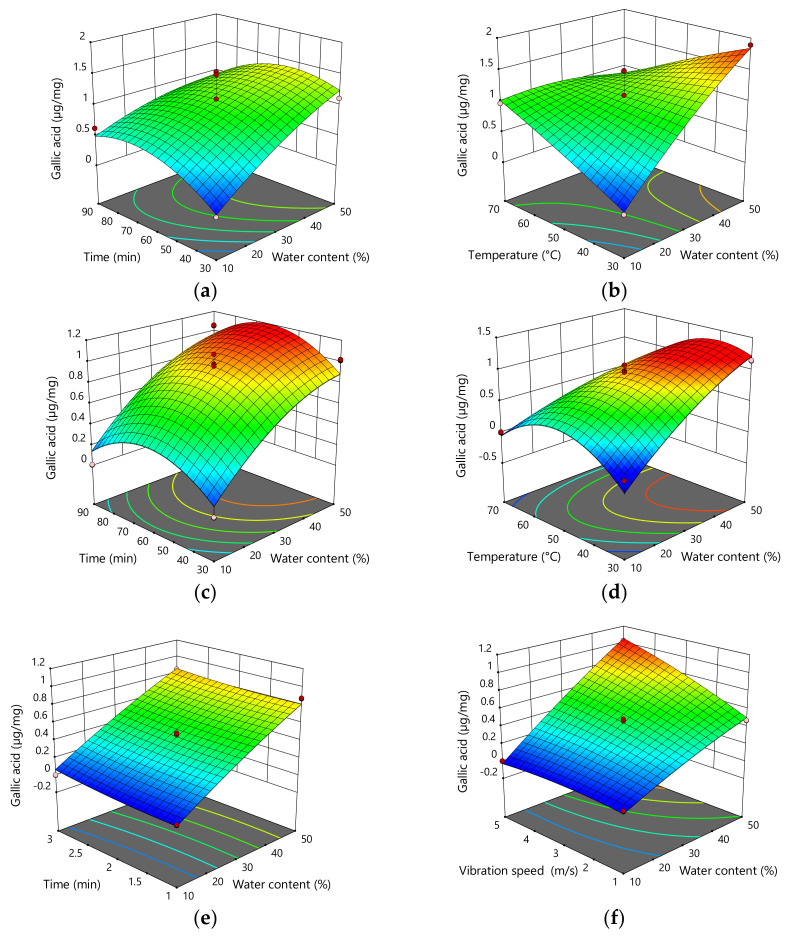
Three-dimensional plots for obtained content of gallic acid as a function of the extraction time, temperature, and H_2_O content for the extraction with mixing and heating (**a**,**b**), UAE (**c**,**d**), and MCE (**e**,**f**).

The addition of water, as well as interactions between the amount of water added and temperature, showed statistically significant influence on the content of gallic acid (*p* = 0.0008; *p* = 0.0021) in the extracts obtained by stirring and mixing. Therefore, the content of gallic acid increased with increased water content. In the extracts obtained by UAE, water addition and temperature showed a statistically significant influence on the content of gallic acid (*p* = 0.0001; *p* = 0.0040). Therefore, gallic acid content increases with increasing water addition and decreasing extraction temperature. In addition, interactions between the amount of added water and temperature also showed a significant influence in terms of gallic acid content (*p* = 0.0074). In the extracts obtained by MCE, water addition and vibration speed (*p* < 0.0001; *p* = 0.0002), as well as the interactions between water addition and vibration speed (*p* = 0.0015), showed statistically significant influence on the content of gallic acid. From this, it can be seen that the content of gallic increased with higher vibration speed and with higher water addition (Figure 2; Table 4).

**Figure 3:**
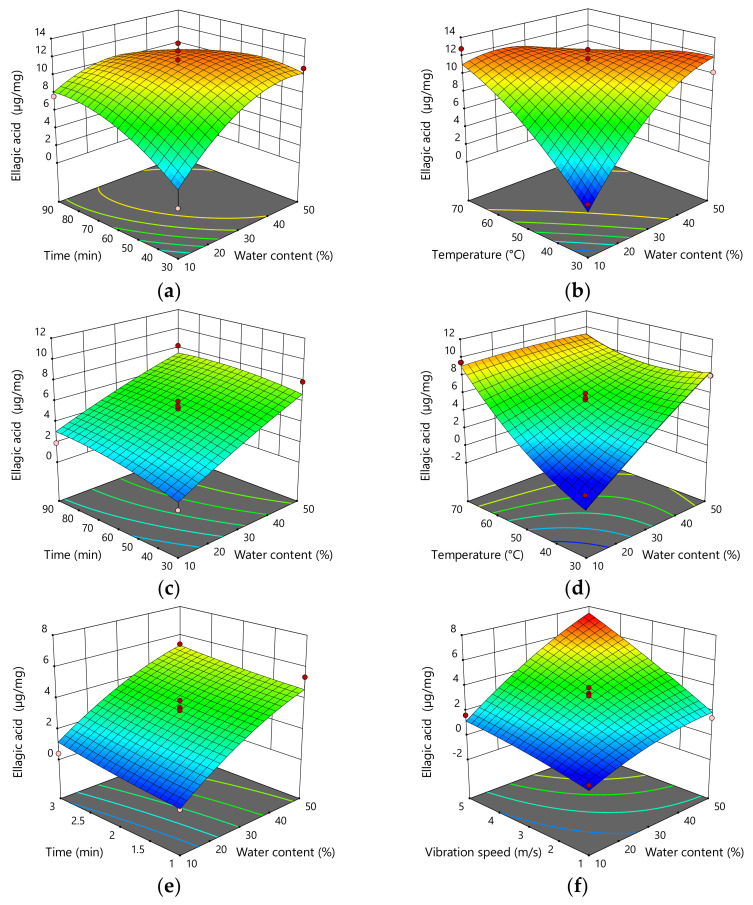
Three-dimensional plots for obtained content of ellagic acid as a function of the extraction time, temperature, and H_2_O content for the extraction with mixing and heating (**a**,**b**), UAE (**c**,**d**), and MCE (**e**,**f**).

The addition of water, as well as interactions between the amount of water added and temperature and interactions between time and temperature, showed statistically significant influence on the content of ellagic acid (*p* = 0.0223; *p* = 0.0029; *p* = 0.0393) in the extracts obtained by stirring and mixing. Therefore, the content of ellagic acid increased with increased water content. In the extracts obtained by UAE, water addition and temperature showed a statistically significant influence on the content of ellagic acid (*p* = 0.0012; *p* = 0.0005). According to that, ellagic acid content increases with increasing water percentage and decreasing extraction temperature. In addition, interactions between the amount of added water and temperature also showed a significant influence in terms of ellagic acid content (*p* = 0.0084). In addition, in the extracts obtained by MCE, water addition and vibration speed (*p* < 0.0001; *p* = 0.0003), as well as the interactions between water percentage and vibration speed (*p* = 0.0349), showed a statistically significant influence on the content of ellagic acid. Therefore, it was observed that the content of ellagic acid increases with the addition of water and vibration speed (Figure 3; Table 5).

**Figure 4:**
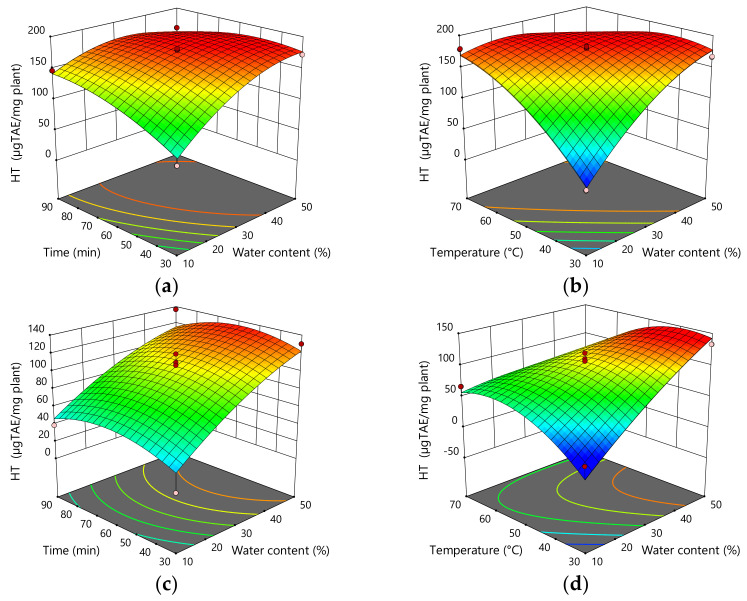

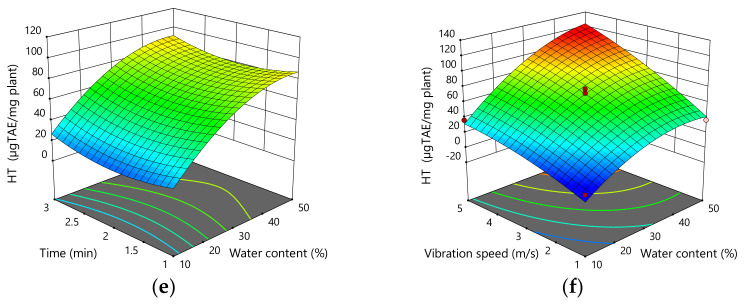
Three-dimensional plots for obtained content of HT as a function of the extraction time, temperature, and H_2_O content for the extraction with mixing and heating (**a**,**b**), UAE (**c**,**d**), and MCE (**e**,**f**).

Water content and temperature showed a statistically significant influence on the content of HT (*p* = 0.0004; *p* = 0.0010) in the extracts obtained by stirring and heating. Therefore, an increase in the amount of water and temperature led to an increase in the content of HT in the extract. In addition, the interaction between water content and temperature, as well as the interaction between time and extraction temperature, had a statistically significant effect on HT content in the extract. On the other hand, in the case of extraction using UAE, HT content in the extract was statistically significantly affected only by water content (*p* = 0.0009), where the amount of HT increased with increasing water content. In addition, HT content was affected by the interaction of water content and extraction temperature. In MCE, the situation was more similar to extraction using stirring and heating, where the content of HT in the extract was statistically significantly influenced by water content and temperature (*p* < 0.0001, *p* < 0.0001). Therefore, an increase in the proportion of water and temperature leads to an increase in the content of HT in the extract. HT content was also statistically significantly affected by the interaction between water content and extraction temperature (Figure 4, Table 6).

Optimization of the extraction process is an important step in selecting significant conditions for achieving the desired yields of certain components in the obtained extract. Using a Box–Behnken design (BBD), second-order response models of the following investigated responses (Table A1) were given, as well as analysis of variance (ANOVA) for the response surface quadratic models for selected responses for the desired components (Table 4, Table 5 and Table 6). According to Table 4, Table 5 and Table 6, it can be seen that all used models for gallic acid, ellagic acid, and HT were statistically significant (*p* < 0.0001–0.1000) with a non-significant lack of fit (*p* = 0.5020–0.7355). R^2^ for gallic acid extraction was 0.9131–0.9864, while for ellagic acid it was 0.8963–0.9440, depending on the extraction technique used. For HT extraction, R^2^ was 0.9003–0.9756 depending on the extraction technique used. According to the above data, the obtained models are adequate for use in the extraction of gallic acid, ellagic acid, and HT using choline chloride:urea (1:2) with three different extraction techniques.

According to RSM, the optimal extraction conditions are those at which the maximum proportion of gallic acid, ellagic acid, and HT is achieved. Depending on the extraction technique used, the optimal extraction conditions differ slightly, as do the extracted amounts of the desired components. According to Table 7, it can be seen that for all three techniques, the addition of water of 50% is the most effective for the extraction of the desired components. Although this is a high percentage of water addition, choline chloride:urea (1:2) has been shown to retain the DES nanostructure until 51 wt% of water [31]. Except for the water content, the optimal extraction temperature was the same for stirring and heating and UAE, which is low and almost room temperature (30 °C), which means that this part can be compared with MCE that takes place at room temperature. The optimal extraction time is longer for stirring and heating compared to the UAE. For MCE, the extraction times are generally lower, so they are difficult to compare with the other two techniques. According to the desirability, it was noticeable that it ranged between 0.917–1.000, which speaks in favor of the obtained model and optimal conditions. In support of the developed model, the obtained amount of gallic acid, ellagic acid, and HT were very similar to the predicted values. In addition, according to the obtained optimal amounts of the desired compounds, it was observed that the extraction obtained by stirring and heating is the most suitable since the highest amounts of gallic acid, ellagic acid, and HT were extracted.

### 2.4. Comparison with Other Extraction Methods

According to the available literature, the most common solid–liquid extraction of *Alchemilla vulgaris* involves the use of methanol, ethanol, ethyl acetate, acetone, and water. In the available literature, the extracts prepared in this way mainly showed biological activity [13,32,33]. In the work of Vlaisavljević et al. [1], it was shown that gallic acid was extracted only with ethyl acetate in the amount of 2465.79 ± 0.01 µg mg^−1^, while with ethanol, methanol, and water no gallic acid was detected in the extract. Data for ellagic acid as well as for HT have not been found in the literature.

Since ethanol can be classified as a bio-solvent, ethanol and aqueous ethanol solutions (30–70% (*v*/*v*)) were chosen to test the effectiveness of the extraction of gallic acid, ellagic acid and HT. In addition, the use of methanol was also examined due to its most common use in the extraction process of bioactive components. According to the results, it is observed that for gallic acid water is the most effective as the solvent at 30 °C for 90 min. Under these conditions a higher amount of gallic acid was extracted (2.21 µg mg^−1^) than under optimal conditions with choline chloride:urea (1:2) using stirring and heating (1.84 µg mg^−1^). On the other hand, the highest amount of ellagic acid was extracted using 30% ethanol (*v*/*v*) at 70 °C for 30 min (7.44 µg mg^−1^), which is less than under optimal conditions with choline chloride:urea (1:2) using stirring and heating (12.08 µg mg^−1^). However, the biggest difference occurs in HT extraction using choline chloride:urea (1:2) and conventional solvents. The highest amount of HT using conventional solvents was achieved with 50% ethanol (*v*/*v*) at 50 °C for 60 min (120.13 µg mg^−1^), which is significantly less than under optimal conditions with choline chloride:urea (1:2) using stirring and heating (178.02 µg mg^−1^) (Table 8). Compared with the other two techniques, it can be seen that by applying UAE and MCE at optimal conditions with choline chloride:urea (1:2), a higher amount of HT was achieved than by the extraction with other solvents which speaks in favor of the effectiveness of DES. Even with the application of MCE in the period of 1.41 min with choline chloride:urea (1:2) as the solvent, a higher amount of HT was achieved than with the use of other solvents in the period up to 90 min.

## 3. Materials and Methods

### 3.1. Chemicals

The standard compounds gallic acid (≥98.0%) (Phytolab GmbH & Co. KG. Vestenbergsgreuth, Germany), ellagic acid (≥95.0%) (Sigma Chemical Co., St. Louis, MO, USA), and tannic acid (95%) (Acros Organics, Geel, Antwerp, Belgium) were used for the chemical analyses. All solvents and chemicals used were of analytical grade.

### 3.2. Plant Material

The dried lady’s mantle plant (*Alchemilla vulgaris* L.), was obtained in spring 2018 from herbal pharmacy Vextra d.o.o. (Mostar, Bosnia and Herzegovina). Before the extraction process, the dried plant was grounded and sieved using a vertical vibratory sieve shaker (LabortechnikGmbh, Ilmenau, Germany) as described in the paper by Jokić et al. [34].

### 3.3. Preparation of DESs

The choline chloride-based DESs were prepared as described in our previously published paper [32]. In this study, seventeen different choline chloride-based DESs were prepared using inexpensive components as shown in Table 1.

### 3.4. Extraction of Desired Components with DESs

The extraction procedure of components from dried lady’s mantle plant is the same as described in our paper [25,35]. Grounded dried lady’s mantle plant (50 mg) was mixed with 1 mL of the solvent or a mixture of DESs with ultrapure H_2_O (Millipore Simplicity 185, Darmstadt, Germany) in certain volume ratios. Screening was performed with all 17 prepared DESs and water (10, 30, and 50% (*v*/*v*)) at 50 °C for 60 min.

After screening, the influence of extraction technique and parameters on the component content in the extracts was examined. The prepared samples were mixed at 1500 rpm in an aluminum block (Stuart SHB) on a magnetic stirrer or in a temperature-controlled ultrasonic bath (Elma P70 H, Singen, Germany) set at 37 Hz and power at 50 W for a period of time and on a certain temperature (Table 2). In addition to the magnetic stirrer and ultrasound, the effect of a BeadRuptor 12 ball mill (Omni International, Kennesaw, GA, USA) for the plant extraction (50 mg of plant + 1 g of glass beads + 1 mL of solvent) at room temperature (24–28 °C) was also examined. The extraction for all three techniques was done according to the parameters in Table 2 and Table 3.

### 3.5. Chemical Characterization of the Extracts

HPLC analyses of gallic and ellagic acids was performed on an Agilent 1260 Infinity II (Agilent, Santa Clara, CA, USA) with chromatographic separation obtained on a ZORBAX Eclipse Plus C18 (Agilent, Santa Clara, CA, USA) column (4.6 × 100 mm, 5 µm). The separation was achieved by gradient elution at a flow rate of 2 mL min^−1^, with gradient elution for 55 min, where 0.25% H_3_PO_4_ and 1.5 % tetrahydrofuran (in millipore water) was used as phase A and methanol was used as phase B. The gradient was set as follows: 0–5 min: 100% A; 5–10 min: 100–85% A; 10–20 min: 85–70% A; 20–40 min: 70–50% A; 40–45 min: 50% A; 45–47 min: 50–0% A; 47–55 min; 0% A. Injection volume was 35 μL, UV detection wavelength 220 and 270 nm and the analysis was performed at room temperature (25 °C). Gallic acid and ellagic acid identification was performed based on the retention time and comparison of the absorption spectrum in the extracts with the spectrum of standard. Quantification was made based on external calibration. The retention time of gallic acid for this method was 2.693 min and for ellagic acid was 24.829 min. Standard stock solutions for gallic acid and ellagic acid were prepared in methanol and calibration was obtained at seven concentrations (concentration range 10.0, 20.0, 50.0, 75.00, 100.0, 200.0, and 500.0 mgL^−1^). Linearity of the calibration curve was confirmed by R^2^ = 0.99964 for gallic acid and R^2^ = 0.99909 for ellagic acid. The results of the content in the analyzed samples were expressed in µg mg^−1^.

Hydrolyzable tannins content was determined spectrophotometrically with the potassium iodate assay described in Rhazi et al. [36]. Five milliliters of aqueous potassium iodide solution (2.5% *w/v*) was heated to 30 °C in a water bath for 7 min, after which 1 mL of the sample (diluted to 10 mgmL^−1^) was added. The mixture was placed in a water bath at 30 °C for 2 min after which the adsorption was measured at 550 nm. The calibration curve was prepared using a tannic acid solution in the range of 20–2500 µgmL^−1,^ and the results were expressed as micrograms of tannic acid equivalent (TAE) per mg of plant (µg of TAE/mg of plant).

### 3.6. Statistical Experimental Design

BBD, explained previously by Bas and Boyaci [37], was employed to determine the optimal extraction conditions under which the highest content of gallic and ellagic acids as well as the tannins is achieved. The independent variables depended on the technique used, water content (X_1_), time (X_2_) and temperature (X_3_), and vibration speed (X_3_) were used in the design. Design-Expert^®^ Commercial Software (ver. 9. Stat-Ease Inc., Minneapolis, MN, USA) was used to analyze the obtained results. In addition, the analysis of variance (ANOVA) was used to evaluate the quality of the fitted model, while the test of statistical difference was based on the total error criteria with a confidence level of 95.0%.

## 4. Conclusions

In this study, the most suitable DESs were chosen, followed by the selection of the most suitable extraction technique as well as the optimal conditions for the extraction of gallic acid, ellagic acid, and HT. Among the examined 17 DESs, choline chloride:urea (1:2) was selected for further optimization with regard to not only the achieved high yields of gallic acid, ellagic acid, and HT, but also to the characteristics of the solvent itself. The content of the desired components is the highest in the extracts obtained by stirring and heating in comparison to UAE and MCE. The influence of a certain percentage of water, extraction time, and temperature (for stirring and heating and for UAE), the addition of a certain percentage of water, extraction time, and vibration speed for MCE on the content of targeted compounds was investigated. The obtained model was tested by comparing the obtained optimal values according to RSM with the experimentally obtained values, which can confirm the effectiveness of the model. The comparison of the effectiveness of choline chloride:urea (1:2) was tested by the comparison with common extraction solvents such as ethanol, H_2_O, aqueous solutions of ethanol (30–70% (*v*/*v*)) and methanol using the same extraction conditions. Thus, choline chloride:urea (1:2) was shown to be a more efficient solvent for the extraction of ellagic acid and HT, while for the extraction of gallic acid H_2_O was a more efficient solvent and then choline chloride:urea (1:2). Given the high proportion of HT in the obtained DES extract in further research, it would be useful to examine the stability of HT over a certain period of time as well as the biological activities of the obtained extracts.

## Figures and Tables

**Table 1:** List of prepared deep eutectic solvents (DESs).

Name	Combination	Molar Ratio
ChClU	Choline chloride:urea	1:2
ChClmU	Choline chloride:N-methylurea	1:3
ChCltU	Choline chloride:thiourea	1:2
ChClG	Choline chloride:glucose	1:1
ChClF	Choline chloride:fructose	1:1
ChClXy	Choline chloride:xylitol	1:1
ChClS	Choline chloride:sorbitol	1:1
ChClB	Choline chloride:butane-1,4-diol	1:2
ChClE	Choline chloride:ethane-1,2-diol	1:2
ChClGl	Choline chloride:glycerol	1:2
ChClA	Choline chloride:acetamide	1:2
ChClM	Choline chloride:malic acid	1:1
ChClC	Choline chloride:citric acid	1:1
ChClMaA	Choline chloride:malonic acid	1:1
ChClOA	Choline chloride:oxalic acid	1:1
ChClLA	Choline chloride:lactic acid	1:2
ChClLeA	Choline chloride:levulinic acid	1:1

**Table 2:** Experimental matrix and obtained values of selected responses (µg mg^−1^ of the plant material) for the extraction with choline chloride:urea (1:2 molar ratio) obtained by extraction of stirring with heating and by UAE.

Run	H_2_O (%)	Time (min)	Temperature (°C)	DES-MIXING			DES-UAE		
				Gallic Acid (µg mg^−1^)	Elagic Acid (µg mg^−1^)	HT (µg TAEmg^-1^)	Gallic Acid (µg mg^−1^)	Elagic Acid (µg mg^−1^)	HT (µgTAE mg^−1^)
1	30	60	50	1.11 ± 0.01	10.75 ± 0.017	171.73 ± 5.03	1.01 ± 0.01	7.89 ± 0.45	130.26 ± 1.60
2	10	90	50	0.94 ± 0.09	10.57 ± 0.12	178.86 ± 0.35	0.91 ± 0.02	4.92 ± 0.06	90.59 ± 1.70
3	10	60	70	0.00	0.74 ± 0.01	69.59 ± 1.03	0.00	0.39 ± 0.55	18.53 ± 4.67
4	30	60	50	1.49 ± 0.02	10.33 ± 0.15	180.06 ± 2.55	0.95 ± 0.01	5.30 ± 0.84	102.06 ± 2.55
5	30	60	50	0.97 ± 0.04	9.94 ± 0.02	165.66 ± 3.99	1.05 ± 0.05	8.21 ± 0.77	136.39 ± 6.27
6	10	30	50	0.97 ± 0.05	12.76 ± 0.13	178.33 ± 0.23	0.00	9.46 ± 0.01	66.59 ± 2.66
7	30	30	70	1.10 ± 0.01	11.69 ± 0.02	181.26 ± 159	1.06 ± 0.09	5.54 ± 0.25	106.73 ± 2.05
8	50	90	50	0.58 ± 0.08	4.98 ± 0.32	96.19 ± 1.42	0.24 ± 0.07	2.31 ± 0.85	50.73 ± 1.72
9	30	90	70	1.00 ± 0.03	10.67 ± 0.16	177.79 ± 1.22	0.97 ± 0.03	6.03 ± 0.12	109.93 ± 1.03
10	10	60	30	0.62 ± 0.00	7.64 ± 0.07	146.53 ± 4.88	0.00	1.91 ± 0.16	38.79 ± 2.00
11	50	60	70	0.91 ± 0.09	10.46 ± 0.51	178.86 ± 1.40	0.91 ± 0.04	5.32 ± 0.18	107.33 ± 0.70
12	50	30	50	0.48 ± 0.04	7.03 ± 0.44	135.13 ± 0.31	0.00	10.87 ± 0.75	37.53 ± 1.15
13	30	60	50	1.03 ± 0.01	11.43 ± 0.14	178.06 ± 3.14	0.00	8.19 ± 0.31	78.53 ± 3.11
14	50	60	30	0.00	1.04 ± 0.43	32.06 ± 2.27	0.00	0.08 ± 0.00	14.73 ± 1.89
15	30	60	50	0.93 ± 0.06	9.59 ± 0.28	147.86 ± 5.02	0.44 ± 0.05	3.15 ± 0.08	64.93 ± 1.86
16	30	90	30	1.89 ± 0.05	10.20 ± 0.49	166.26 ± 2.88	1.14 ± 0.05	7.99 ± 0.29	132.99 ± 3.32
17	30	30	30	0.90 ± 0.02	6.01 ± 59	140.99 ± 3.56	0.00	7.67 ± 0.22	42.79 ± 2.23

Results are expressed as mean value ± standard deviation (*n* = 3).

**Table 3:** Experimental matrix and obtained values of selected responses (µg mg^−1^ of the plant material) for the extraction with choline chloride:urea (1:2 molar ratio) obtained by MCE.

Run	H_2_O (%)	Time (min)	Vibration Speed (m s^−1^)	Gallic Acid (µg mg^−1^)	Elagic Acid (µg mg^−1^)	HT (µgTAE mg^−1^)
1	30	3	1	0.43 ± 0.00	3.42 ± 0.02	72.26 ± 0.42
2	30	2	3	0.00 ± 0.00	0.39 ± 0.02	21.59 ± 1.42
3	50	3	3	0.46 ± 0.05	3.89 ± 0.48	65.19 ± 4.52
4	50	1	3	0.00 ± 0.00	0.00 ± 0.00	3.06 ± 3.40
5	50	2	5	1.02 ± 0.02	7.07 ± 0.44	115.26 ± 1.06
6	10	3	3	0.50 ± 0.04	4.05 ± 0.06	106.79 ± 2.66
7	50	2	1	0.43 ± 0.00	3.42 ± 0.01	73.73 ± 0.31
8	10	2	1	0.41 ± 0.00	3.24 ± 0.01	70.73 ± 1.10
9	10	1	3	0.48 ± 0.01	2.19 ± 0.00	72.19 ± 4.97
10	30	1	5	0.22 ± 0.07	0.67 ± 0.04	35.26 ± 2.31
11	30	2	3	0.00 ± 0.00	1.66 ± 0.01	36.73 ± 1.70
12	10	2	5	0.00 ± 0.00	0.14 ± 0.02	22.73 ± 0.95
13	30	2	3	0.63 ± 0.12	5.68 ± 0.02	103.79 ± 1.89
14	30	2	3	0.87 ± 0.04	5.38 ± 0.05	105.06 ± 5.79
15	30	1	1	0.47 ± 0.07	1.44 ± 0.03	36.99 ± 1.86
16	30	2	3	0.36 ± 0.02	2.14 ± 0.05	50.39 ± 0.90
17	30	3	5	0.84 ± 0.06	0.84 ± 0.09	103.79 ± 0.80

Results are expressed as mean value ± standard deviation (*n* = 3).

**Table 4:** Analysis of variance (ANOVA) for the response surface quadratic models for selected responses for gallic acid.

Source	Sum of Squares	df	Mean Square	F-Value	*p*-Value
*Mixing and heating*					
Model	3.12	9	0.3470	8.18	0.0056
*X*_1_	1.34	1	1.34	31.68	0.0008
*X*_2_	0.0098	1	0.0098	0.2309	0.6455
*X*_3_	0.0000	1	0.0000	0.0012	0.9736
*X*_1_ *X*_2_	0.1444	1	0.1444	3.40	0.1076
*X*_1_ *X*_3_	0.9604	1	0.9604	22.63	0.0021
*X*_2_ *X*_3_	0.2025	1	0.2025	4.77	0.0652
*X*_1_^2^	0.0547	1	0.0547	1.29	0.2936
*X*_2_^2^	0.3764	1	0.3764	8.87	0.0206
*X*_3_^2^	0.0049	1	0.0049	0.1147	0.7448
Residual	0.2971	7	0.0424		
Lack of fit	0.0741	3	0.0247	0.4426	0.7355
Pure error	0.2231	4	0.0558		
Cor total	3.42	16			
R^2^ = 0.9131					
*UAE*					
Model	3.64	9	0.4048	17.30	0.0005
*X*_1_	1.28	1	1.28	54.71	0.0001
*X*_2_	0.0072	1	0.0072	0.3077	0.5963
*X*_3_	0.4141	1	0.4141	17.70	0.0040
*X*_1_ *X*_2_	0.0004	1	0.004	0.0171	0.8996
*X*_1_ *X*_3_	0.3249	1	0.3249	13.89	0.0074
*X*_2_ *X*_3_	0.0100	1	0.0100	0.4274	0.5341
*X*_1_^2^	0.1038	1	0.1038	4.44	0.0732
*X*_2_^2^	0.3115	1	0.3115	13.31	0.0082
*X*_3_^2^	1.06	1	1.06	45.35	0.0003
Residual	0.1638	7	0.0234		
Lack of fit	0.1355	3	0.0452	6.38	0.0527
Pure error	0.0283	4	0.0071		
Cor total	3.81	16			
R^2^ = 0.9570					
*MCE*					
Model	1.53	9	0.1695	56.24	<0.0001
*X*_1_	1.28	1	1.28	424.64	<0.0001
*X*_2_	0.0072	1	0.0072	2.39	0.1661
*X*_3_	0.1512	1	0.1512	50.18	0.0002
*X*_1_ *X*_2_	0.0002	1	0.0002	0.0746	0.796
*X*_1_ *X*_3_	0.0756	1	0.0756	25.09	0.0015
*X*_2_ *X*_3_	0.0000	1	0.0000	0.0083	0.9300
*X*_1_^2^	0.0048	1	0.0048	1.59	0.2476
*X*_2_^2^	0.0019	1	0.0019	0.6308	0.4532
*X*_3_^2^	0.0048	1	0.0048	1.59	0.2476
Residual	0.0211	7	0.0030		
Lack of fit	0.0173	3	0.0058	6.07	0.0570
Pure error	0.0038	4	0.0009		
Cor total	1.55	16			
R^2^ = 0.9864					

*X*_1_, water content (%); *X*_2_, time (min); *X*_3_, temperature (°C); *p* < 0.01 highly significant; 0.01 ≤ *p* < 0.05 significant; *p* ≥ 0.05 not significant.

**Table 5:** Analysis of variance (ANOVA) for the response surface quadratic models for selected responses for ellagic acid.

Source	Sum of Squares	df	Mean Square	F-Value	*p*-Value
*Mixing and heating*					
Model	191.87	9	21.32	6.72	0.0100
*X*_1_	27.08	1	27.08	8.54	0.0223
*X*_2_	4.96	1	4.96	1.56	0.2513
*X*_3_	16.30	1	16.30	5.14	0.0578
*X*_1_ *X*_2_	14.86	1	14.86	4.68	0.0672
*X*_1_ *X*_3_	63.28	1	63.28	19.95	0.0029
*X*_2_ *X*_3_	20.30	1	20.30	6.40	0.0393
*X*_1_^2^	22.78	1	22.78	7.18	0.0316
*X*_2_^2^	10.39	1	10.39	3.27	0.1133
*X*_3_^2^	7.51	1	7.51	2.37	0.1677
Residual	22.21	7	3.17		
Lack of fit	18.23	3	6.08	6.11	0.0565
Pure error	3.98	4	0.9950		
Cor total	214.08	16			
R^2^ = 0.8963					
*UAE*					
Model	149.79	9	16.64	9.31	0.0038
*X*_1_	49.60	1	49.60	27.74	0.0012
*X*_2_	3.59	1	3.59	2.01	0.1993
*X*_3_	64.18	1	64.18	35.90	0.0005
*X*_1_ *X*_2_	0.3600	1	0.3600	0.2014	0.6672
*X*_1_ *X*_3_	23.52	1	23.52	13.16	0.0084
*X*_2_ *X*_3_	0.8464	1	0.8464	0.4734	0.5136
*X*_1_^2^	0.2345	1	0.2345	0.1312	0.7279
*X*_2_^2^	0.6940	1	0.6940	0.3882	0.5530
*X*_3_^2^	7.05	1	7.05	3.94	0.0874
Residual	1.79	7	1.79		
Lack of fit	3.43	3	3.43	6.21	0.0550
Pure error	0.5531	4	0.5531		
Cor total		16			
R^2^ = 0.9229					
*MCE*					
Model	68.29	9	7.59	13.12	0.0013
*X*_1_	36.42	1	36.42	62.97	<0.0001
*X*_2_	1.39	1	1.39	2.41	0.1644
*X*_3_	25.24	1	25.24	43.64	0.0003
*X*_1_ *X*_2_	0.0169	1	0.0169	0.0292	0.8691
*X*_1_ *X*_3_	3.94	1	3.94	6.81	0.0349
*X*_2_ *X*_3_	0.0064	1	0.0064	0.0111	0.9192
*X*_1_^2^	1.06	1	1.06	1.84	0.2175
*X*_2_^2^	0.0343	1	0.0343	0.0593	0.8146
*X*_3_^2^	0.1476	1	0.1476	0.2553	0.6289
Residual	4.05	7	0.5784		
Lack of fit	2.46	3	0.8197	2.06	0.2478
Pure error	1.59	4	0.3974		
Cor total	72.34	16			
R^2^ = 0.9440					

*X*_1_, water content (%); *X*_2_, time (min); *X*_3_, temperature (°C); *p* < 0.01 highly significant; 0.01 ≤ *p* < 0.05 significant; *p* ≥ 0.05 not significant.

**Table 6:** Analysis of variance (ANOVA) for the response surface quadratic models for selected responses for HT.

Source	Sum of Squares	df	Mean Square	F-Value	*p*-Value
*Mixing and heating*					
Model	28,766.77	9	3196.31	20.79	0.0003
*X*_1_	5947.59	1	5947.59	38.68	0.0004
*X*_2_	792.22	1	792.22	5.15	0.0575
*X*_3_	4519.15	1	4519.15	29.39	0.0010
*X*_1_ *X*_2_	1722.67	1	1722.67	11.20	0.0123
*X*_1_ *X*_3_	7356.49	1	7356.49	47.84	0.0002
*X*_2_ *X*_3_	22,237.29	1	22,237.29	14.55	0.0066
*X*_1_^2^	2454.50	1	2454.50	15.96	0.0052
*X*_2_^2^	854.31	1	854.31	5.56	0.0506
*X*_3_^2^	2268.57	1	2268.57	14.75	0.0064
Residual	1076.42	7	153.77		
Lack of fit	893.18	3	297.73	6.50	0.0511
Pure error	183.24	4	45.81		
Cor total	29,843.19	16			
R^2^ = 0.9639					
*UAE*					
Model	23,803.68	9	2644.85	7.02	0.0088
*X*_1_	11,536.05	1	11,536.05	30.62	0.0009
*X*_2_	0.0210	1	0.0210	0.0001	0.9942
*X*_3_	179.93	1	179.93	0.4776	0.5118
*X*_1_ *X*_2_	49.91	1	49.91	0.1325	0.7266
*X*_1_ *X*_3_	5045.26	1	5045.26	13.39	0.0081
*X*_2_ *X*_3_	761.76	1	761.76	2.02	0.1960
*X*_1_^2^	356.01	1	356.01	0.9451	0.3634
*X*_2_^2^	1016.84	1	1016.84	2.70	0.1444
*X*_3_^2^	4381.32	1	4381.32	11.63	0.0113
Residual	2636.92	7	37670		
Lack of fit	2190.64	3	730.21	6.54	0.0506
Pure error	446.29	4	111.57		
Cor total	26,440.60	16			
R^2^ = 0.9003					
*MCE*					
Model	18,998.68	9	2110.96	31.06	<0.0001
*X*_1_	9590.43	1	9590.43	141.10	<0.0001
*X*_2_	15.10	1	15.10	0.2221	0.6518
*X*_3_	7088.83	1	7088.83	104.30	<0.0001
*X*_1_ *X*_2_	0.0042	1	0.0042	0.0001	0.9939
*X*_1_ *X*_3_	497.29	1	497.29	7.32	0.0304
*X*_2_ *X*_3_	71.06	1	71.06	1.05	0.3406
*X*_1_^2^	1266.92	1	1266.92	18.64	0.0035
*X*_2_^2^	342.48	1	342.48	5.04	0.0597
*X*_3_^2^	165.20	1	165.20	2.43	0.1630
Residual	475.78	7	67.97		
Lack of fit	395.57	3	131.86	6.58	0.0502
Pure error	80.20	4	20.05		
Cor total	19,474.45	16			
R^2^ = 0.9756					

X_1_, water content (%); X_2_, time (min); X_3_, temperature (°C); *p* < 0.01 highly significant; * 0.01 ≤ *p* < 0.05 significant; *p* ≥ 0.05 not significant.

**Table 7:** Optimal extraction parameters for each investigated response obtained by RSM.

Optimal Parameters and Results	Extraction Method		
	Stirring and Heating	Ultrasound-Assisted Extraction	Mechanochemical Extraction
Extraction parameters	50% H_2_O 68.2 min 30 °C	50% H_2_O 56.6 min 30 °C	49.5% H_2_O 1.41 min 4.99 m/s
Predicted gallic acid content (µg/mg)	1.84	1.18	1.03
Predicted ellagic acid content (µg/mg)	12.08	8.39	7.18
Predicted HT content (µg TAE/mg)	178.015	141.18	127.99
Desirability	0.965	0.917	1.000
Obtained gallic acid content (µg/mg)	1.91 ± 0.04	1.19 ± 0.01	1.04 ± 0.00
Obtained ellagic acid content (µg/mg)	12.21 ± 0.09	8.18 ± 0.07	7.10 ± 0.12
Obtained HT content (µg TAE/mg)	169.07 ± 3.42	143.24 ± 2.24	132.26 ± 1.89

Results are expressed as mean value ± standard deviation (*n* = 3).

**Table 8:** Determined values of selected responses (µg mg^−1^ of the plant material) for the extraction in extracts obtained with different solvents by stirring with heating (*n* = 3).

Solvent	Time (min)	Temperature (°C)	GA (µg mg^−1^)	EA (µg mg^−1^)	HT (µgTAE mg^−1^)
30% ethanol (*v*/*v*)	30	30	0.56 ± 0.02	3.44 ± 0.33	90.56 ± 3.18
	60		0.56 ± 0.03	3.34 ± 0.09	81.73 ± 3.31
	90		0.61 ± 0.00	3.65 ± 0.07	93.66 ± 2.80
	30	50	0.52 ± 0.02	3.08 ± 0.46	76.33 ± 0.42
	60		0.54 ± 0.00	4.67 ± 0.11	75.26 ± 4.98
	90		0.55 ±0.00	3.62 ± 0.15	88.86 ± 2.12
	30	70	0.79 ± 0.01	7.44 ± 0.18	92.39 ± 2.95
	60		0.57 ± 0.00	3.71 ± 0.02	71.53 ± 0.70
	90		0.53 ± 0.00	3.68 ± 0.09	97.39 ± 1.10
50% ethanol (*v*/*v*)	30	30	0.77 ± 0.08	3.05 ± 0.19	92.66 ± 6.77
	60		0.49 ± 0.00	3.23 ± 0.04	84.19 ± 1.50
	90		0.53 ± 0.04	3.49 ± 0.06	98.59 ± 2.00
	30	50	0.72 ± 0.02	3.15 ± 0.36	88.19 ± 2.30
	60		0.53 ± 0.04	3.92 ± 0.28	120.13 ± 2.58
	90		0.51 ± 0.01	3.94 ± 0.03	99.06 ± 1.91
	30	70	0.57 ± 0.00	4.64 ± 0.03	100.39 ± 2.58
	60		0.53 ± 0.00	3.95 ± 0.24	62.19 ± 3.23
	90		0.50 ± 0.00	3.56 ± 0.05	90.33 ± 3.14
70% ethanol (*v*/*v*)	30	30	0.55 ± 0.09	1.22 ± 0.05	72.46 ± 1.64
	60		0.51 ± 0.01	1.81 ± 0.34	47.93 ± 0.61
	90		0.52 ± 0.01	2.40 ± 0.10	51.93 ± 2.64
	30	50	0.54 ± 0.00	2.97 ± 0.01	35.79 ± 1.20
	60		0.51 ± 0.00	3.07 ± 0.02	62.26 ± 0.60
	90		0.54 ± 0.00	3.58 ± 0.18	51.53 ± 2.80
	30	70	0.61 ± 0.00	4.41 ± 0.05	49.46 ± 2.94
	60		0.60 ± 0.04	4.36 ±0.47	77.33 ± 2.23
	90		0.55 ± 0.00	3.83 ± 0.19	71.79 ± 2.67
ethanol	30	30	-	0.19 ± 0.19	0.46 ± 0.03
	60		0.41 ± 0.00	0.43 ± 0.00	0.86 ± 0.20
	90		0.41 ± 0.01	0.69 ± 0.16	3.86 ± 0.51
	30	50	0.50 ± 0.03	0.90 ± 0.14	0.66 ± 0.08
	60		0.67 ± 0.00	2.44 ± 0.01	2.19 ± 0.04
	90		-	2.48 ± 0.30	2.73 ± 0.07
	30	70	-	2.16 ± 0.03	1.26 ± 0.08
	60		-	3.73 ± 0.35	29.73 ± 0.09
	90		-	4.03 ± 0.29	26.79 ± 0.42
methanol	30	30	1.11 ± 0.03	1.31 ± 0.19	35.46 ± 0.53
	60		0.61 ± 0.05	1.68 ± 0.05	-
	90		0.65 ± 0.04	2.14 ± 0.20	40.79 ± 1.40
	30	50	0.68 ± 0.02	1.95 ± 0.13	41.33 ± 3.28
	60		0.69 ± 0.04	2.19 ± 0.20	46.93 ± 4.15
	90		0.54 ± 0.05	3.90 ± 0.05	81.13 ± 4.10
	30	70	1.04 ± 0.04	4.24 ± 0.01	-
	60		1.03 ± 0.17	5.38 ± 0.63	49.33 ± 3.52
	90		0.62 ± 0.00	3.55 ± 0.01	29.66 ± 0.40
water	30	30	2.19 ± 0.02	6.94 ± 0.07	60.13 ± 2.31
	60		2.02 ± 0.02	6.42 ± 0.31	50.33 ± 0.05
	90		2.21 ± 0.12	7.54 ± 0.01	86.06 ± 3.42
	30	50	1.47 ± 0.05	3.32 ± 0.43	43.93 ± 3.52
	60		2.03 ±0.05	4.38 ± 0.12	41.66 ± 3.30
	90		1.93 ± 0.03	3.88 ± 0.06	39.39 ± 3.80
	30	70	1.03 ± 0.05	8.24 ± 0.32	68.89 ± 2.84
	60		1.17 ± 0.09	8.17 ± 0.72	67.93 ± 3.69
	90		0.64 ± 0.04	4.08 ± 0.11	90.39 ± 1.40

## Data Availability

The data presented in this study are available from the authors.

## Appendix A

**Table A1:** Second-order response models for the following investigated responses (coded Equations).

Strirring and heating	Y*_Gallic acid_*= 1.088 + 0.41*X*_1_ + 0.035*X*_2_ − 0.0025*X*_3_ − 0.19 *X*_1_*X*_2_ − 0.49 *X*_1_*X*_3_ − 0.225*X*_2_*X*_3_ − 0.114*X*_1_^2^ − 0.299*X*_2_^2^ − 0.034*X*_3_^2^	(A1)
	Y*_Ellagic acid_* = 11.164 + 1.84*X*_1_ + 0.7875*X*_2_ + 1.4275*X*_3_ − 1.9275 *X*_1_*X*_2_ − 3.9775*X*_1_*X*_3_ − 2.2525*X*_2_*X*_3_ −2.32575*X*_1_^2^ − 1.57075*X*_2_^2^ − 1.33575*X*_3_^2^	(A2)
	Y*_HT_* = 176.766 + 27.2662*X*_1_ + 9.95125*X*_2_ + 23.7675*X*_3_+ − 20.7525 *X*_1_*X*_2_ − 42.885*X*_1_*X*_3_ − 23.65*X*_2_*X*_3_ − 24.1443*X*_1_^2^ − 14.2443*X*_2_^2^ − 23.2117*X*_3_^2^	(A3)
Ultrasound-assisted extraction	Y*_Gallic acid_*= 0.944 + 0.4*X*_1_ + 0.03*X*_2_ − 0.2275*X*_3_ + 0.01*X*_1_*X*_2_ − 0.285*X*_1_*X*_3_ − 0.05*X*_2_*X*_3_ − 0.157*X*_1_^2^ − 0.272*X*_2_^2^ − 0.502*X*_3_^2^	(A4)
	Y*_Ellagic acid_* = 5.242 + 2.49*X*_1_ + 0.67*X*_2_ + 2.8325*X*_3_ − 0.3*X*_1_*X*_2_ − 2.425*X*_1_*X*_3_ + 0.46*X*_2_*X*_3_ − 0.236*X*_1_^2^ − 0.406*X*_2_^2^ + 1.294*X*_3_^2^	(A5)
	Y*_HT_* = 105.728 + 37.9738*X*_1_ − 0.05125*X*_2_ − 4.7425*X*_3_ − 3.5325*X*_1_*X*_2_ − 35.515*X*_1_*X*_3_ − 13.8 *X*_2_*X*_3_ − 9.19525*X*_1_^2^ − 15.5403*X*_2_^2^ − 32.2578*X*_3_^2^	(A6)
Mechanochemical extraction	Y*_Gallic acid_*= 0.44 + 0.4*X*_1_ + 0.03*X*_2_ + 0.1375*X*_3_ − 0.0075*X*_1_*X*_2_ + 0.1375*X*_1_*X*_3_ − 0.0025*X*_2_*X*_3_ − 0.03375*X*_1_^2^ + 0.02125*X*_2_^2^ − 0.03375*X*_3_^2^	(A7)
	Y*_Ellagic acid_* = 3.232 + 2.13375*X*_1_ + 0.4175*X*_2_ + 1.77625*X*_3_ − 0.065*X*_1_*X*_2_ + 0.9925*X*_1_*X*_3_ + 0.04*X*_2_*X*_3_ − 0.50225*X*_1_^2^ + 0.09025*X*_2_^2^ − 0.18725*X*_3_^2^	(A8)
	Y*_HT_* = 71.62 + 34.6238*X*_1_ + 1.37375*X*_2_ + 29.7675*X*_3_ − 0.0325*X*_1_*X*_2_ + 11.15*X*_1_*X*_3_ − 4.215*X*_2_*X*_3_ + − 17.3463*X*_1_^2^ + 9.01875*X*_2_^2^ − 6.26375*X*_3_^2^	(A9)
